# Association between Quality of Maternal Prenatal Food Source and Preparation and Breastfeeding Duration in the Environmental Influences on Child Health Outcome (ECHO) Program

**DOI:** 10.3390/nu14224922

**Published:** 2022-11-21

**Authors:** Emily Zimmerman, Kennedy K. Gachigi, Rachel F. Rodgers, Deborah J. Watkins, Megan Woodbury, José F. Cordero, Akram Alshawabkeh, John D. Meeker, Gredia Huerta-Montañez, Zaira Rosario Pabon, Morgan Hines, Carmen M. Velez-Vega, Carlos A. Camargo, Yeyi Zhu, Sara S. Nozadi, Sarah S. Comstock, Christine Hockett, Patrick M. Tarwater, on behalf of program collaborators for Environmental influences on Child Health Outcomes

**Affiliations:** 1 Department of Communication Sciences and Disorders, Northeastern University, 228C Forsyth Building, 360 Huntington Ave, Boston, MA 02115, USA; 2 Department of Epidemiology, Johns Hopkins University, Baltimore, MD 21205, USA; 3 Department of Applied Psychology, Northeastern University, Boston, MA 02115, USA; 4 Department of Psychiatric Emergency & Acute Care, Lapeyronie Hospital, CHRU Montpellier, 34090 Montpellier, France; 5 Department of Environmental Health Sciences, University of Michigan School of Public Health, Ann Arbor, MI 48109, USA; 6 Department of Epidemiology and Biostatistics, University of Georgia, Athens, GA 30602, USA; 7 Department of Civil and Environmental Engineering, Northeastern University, Boston, MA 02115, USA; 8 Department of Electrical and Computer Engineering, Northeastern University, Boston, MA 02115, USA; 9 Department of Social Sciences, University of Puerto Rico, Medical Sciences Campus, San Juan, PR 00936, USA; 10 Department of Emergency Medicine, Massachusetts General Hospital, Boston, MA 02114, USA; 11 Division of Research, Kaiser Permanente Northern California, Oakland, CA 94612, USA; 12 Health Sciences Center, College of Pharmacy, University of New Mexico, Albuquerque, NM 87131, USA; 13 Department of Food Science and Human Nutrition, Michigan State University, East Lansing, MI 48824, USA; 14 Avera Research Institute, Sioux Falls, SD 57108, USA; 15 Department of Pediatrics, University of South Dakota School of Medicine, Sioux Falls, SD 57105, USA; 16 Department of Epidemiology and Biostatistics, Texas A&M University, College Station, TX 77843, USA

**Keywords:** breastfeeding duration, food source, food preparation, pregnancy

## Abstract

This study examined the relationship between maternal food source and preparation during pregnancy and the duration of breastfeeding among 751 mother–child dyads in the United States. The data collected from the Environmental influences on Child Health Outcomes (ECHO) Program included twelve cohorts of mothers (age ≥ 18) who delivered infant(s). Three categories of maternal food source and preparation including, High, Moderate, or Low Food Source Quality were derived from the mother report. The mean duration of breastfeeding differed strongly across the three categories. The High Food Source Quality group breastfed an average of 41 weeks, while shorter durations were observed for the Moderate (26 weeks) and Low (16 weeks) Food Source Quality groups. Cox proportional hazards models were used to estimate the relative hazard of time to breastfeeding cessation for each participant characteristic. The full model adjusted for clustering/cohort effect for all participant characteristics, while the final model adjusted for the subset of characteristics identified from variable reduction modeling. The hazard of breastfeeding cessation for those in the High Food Source Quality group was 24% less than the Moderate group (RH = 0.76; 95% CI, 0.63–0.92). Pregnant women in the High Food Source Quality group breastfed longer than the Moderate and Low groups. We encourage more detailed studies in the future to examine this relationship longitudinally.

## 1. Introduction

Breastfeeding is associated with a range of positive physical and mental health outcomes for mothers and infants, with evidence suggesting that a longer breastfeeding duration is associated with the greatest benefits to the child and the mother [1,2,3]. However, several difficulties may impede breastfeeding initiation [4] or lead to earlier breastfeeding cessation [5,6]. Recent research has established a link between maternal eating post-pregnancy and child eating behaviors [7,8,9]. Accordingly, diet and eating patterns during pregnancy are also likely important elements for the health of the mother–infant dyad and may play a role in breastfeeding behaviors after birth. However, little is known about how prenatal maternal dietary factors may be associated with breastfeeding. This present study aimed to examine the relationship between maternal food source and preparation during pregnancy and breastfeeding outcomes among mother–infant dyads.

Both the World Health Organization and UNICEF recommend that mothers initiate breastfeeding within the first hour of birth and exclusively breastfeed for the first 6 months of life [10]. Although an emerging body of literature has focused on the influence of the maternal diet during the lactation on milk composition and breastfeeding outcomes [11,12], comparatively little work has focused on the relationship between the maternal diet quality during pregnancy and breastfeeding outcomes.

A diet during pregnancy that is comprised of high-quality food, supplying essential macro and micro-nutrients, is crucial to the health status of the mother and the child [13]. However, maternal food sources and food preparation (e.g., organic foods, pre-made foods) are likely heavily determined by socio-economic status and maternal age [14]. Given that economic factors and food insecurity influence the breastfeeding initiation and duration, [15], we may expect those with a diet of lower quality food source and preparation to experience greater barriers to breastfeeding, resulting in shorter breastfeeding duration. Pregnancy and lactation place additional demands on a mother’s body. Optimal quality and preparation of foods consumed to meet those demands may not be available to all. To date, this has not been empirically examined with a large, diverse sample.

One of the few studies in this area investigated the role of maternal diet, personal characteristics, and willingness to breastfeed on breastfeeding duration for 161 hospitalized neonates in Greece. This study found that the adjusted odds ratios for breastfeeding at 6 months were significantly higher (2.15) for women who consumed ≥3.5 servings of fruits/day, as compared to those who consumed fewer fruits and vegetables [16]. These initial findings provide preliminary support for a relationship between diet quality and breastfeeding duration; however, more research is needed in other populations with a larger sample size to clarify the relationship between maternal diet quality during pregnancy and breastfeeding outcomes.

This study examines the relationship between self-reported food source and preparation during pregnancy and breastfeeding duration among mother child–dyads in the United States. These data for this study are derived from the Environmental influences on Child Health Outcomes (ECHO) study, an NIH funded large nationwide collection of maternal–child cohorts [17,18,19]. We hypothesize that poor maternal food source and preparation (e.g., eating a lot of processed foods or takeout) would be associated with a shorter breastfeeding duration, even when accounting for low maternal socioeconomic status and younger age, which are documented predictors of a shorter breastfeeding duration [20].

## 2. Materials and Methods

### 2.1. Study Design

This study utilized data from a large multi-cohort study with self-report and objective measures collected from the parents. This study was conducted in accordance with the Declaration of Helsinki. All participants consented to participate in their local ECHO cohort and for their information to be shared with the ECHO consortium. Both a central and a cohort-specific institutional review board monitored human subject activities at each cohort site and at the centralized ECHO Data Analysis Center. All participants provided informed consent.

### 2.2. Sample

The current study analyzed both extant data, which were collected prior to the formation of ECHO in 2016, and newly collected ECHO cohort data from mothers 18 years of age or older, who delivered either preterm or full-term prior to 31 August 2021. Survey data from children and their mothers were used from twelve of these ECHO cohorts (Supplementary Table S1).

**Figure 1:**
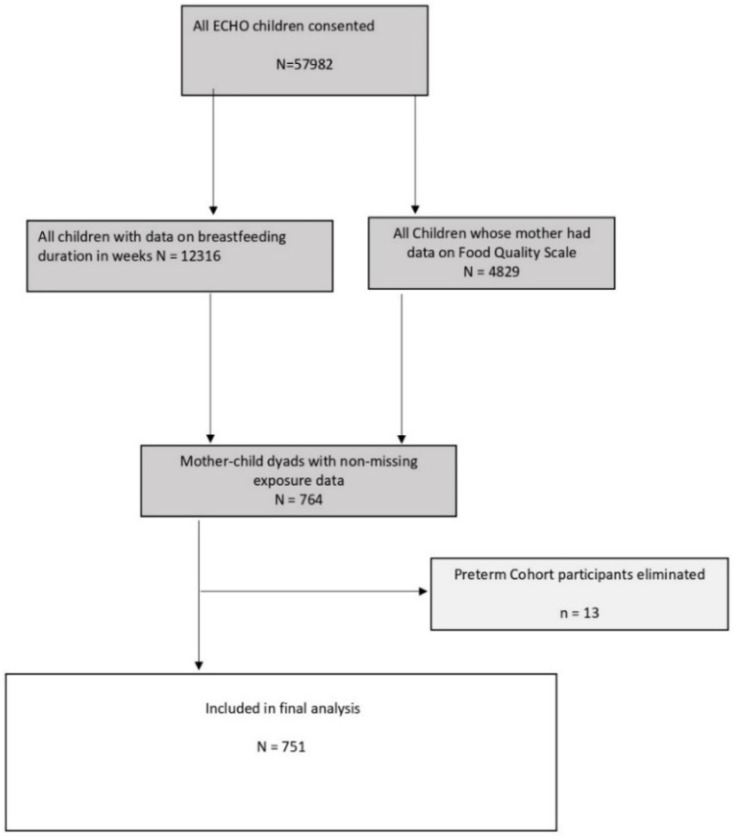
Inclusion in the analysis.

Additional inclusion criteria included self-report data from mothers between 18 and <47 years of age with information regarding maternal food source and the preparation and breastfeeding duration (*n* = 764). One preterm cohort was excluded, as it only enrolled infants from the Neonatal Intensive Care Unit. After exclusions, a total of 751 mother–child dyads across twelve cohorts, enrolled from five U.S. states, met the criteria, and were included in the current analysis (Figure 1).

### 2.3. Measures

#### 2.3.1. Breastfeeding Duration

Duration of breastfeeding, or time to breastfeeding cessation in months, was the outcome variable of interest. For this study, breastfeeding duration data were collected from the ECHO-wide Infant Feeding Practices (IFP) Questionnaire, a self-report tool used to evaluate feeding beliefs and behaviors among mothers of infants [21]. The IFP comprises 39 items on maternal beliefs, 24 items on behaviors, and 20 behavioral items that pertain to solid feeding for infants over 6 months. The current analysis utilized one IFP question to determine the breastfeeding duration: “How old was the child when the child’s biological mother completely stopped breastfeeding and pumping milk?” This variable was utilized after the ECHO-wide data harmonization process for the length of breastfeeding that was completed as of 31 August 2021.The harmonization process uses both new data collected by the standardized protocol and cohort-specific extant data to maximize the sample size and power for analysis. The time to breastfeeding cessation was examined as a continuous variable, and no cutoffs were considered.

#### 2.3.2. Maternal Food Source Quality

Maternal, prenatal Food Source Quality was classified as High, Moderate, or Low based on the mean responses to seven food source-based questions from the ECHO Maternal Food Source and Preparation Questionnaire. This questionnaire was administered to mothers during the prenatal life stage. The Food Source Quality score for each question was recorded and summarized (i.e., arithmetic mean) across the seven questions that captured the self-reported frequency of canned food consumption; fast food or restaurant takeout consumption; cooking at home; and organic vegetables, organic fruits, organic meat and/or poultry, and organic dairy product consumption in the past 30 days (Table 1).

The Food Source Quality mean score, a continuous variable, ranged from 1 to 4. A mean score of 1 indicates ‘Excellent’ food source responses across all seven questions. Inversely, a mean score equal to 4 indicates a response of ‘Poor’ on all questions. For example, a mother who never or rarely ate fast food or packaged food was coded as having an ‘Excellent’ Food Source Quality score (i.e., equal to 1) for that question and, conversely, a mother who ate fast food or packaged food at least once a day was coded as having a ‘Poor’ Food Source Score (i.e., equal to 4). For the same question, responses of 1 to 3 times a week were coded as ‘Average’, and 4 to 6 times a week were coded as ‘Poor.’ This Food Source classification allowed us to capture the maternal responses across a variety of questions. For instance, a mother might report never eating takeout, which would be coded as ‘Excellent’, and report always eating canned food, which would be coded as ‘Poor.’ Thus, our Food Source Quality mean score allowed us to account for mothers who scored ‘Excellent’ on some questions and ‘Poor’ on others within our analyses.

Table 2 shows the categorization of the Food Source Quality mean score into three groups, and Table 3 presents the frequencies and percentages of the covariates across the three Food Source Quality categories. This categorization enabled us to model the effect of Food Source Quality on breastfeeding duration from a survival analysis perspective.

#### 2.3.3. Participant Characteristics

Demographic and socio-economic characteristics of participants considered in the analysis were maternal race/ethnicity, maternal education, household income, marital status, maternal depression diagnosis during pregnancy, parity, maternal age at birth, maternal pre-pregnancy body mass index (BMI), child gestational age at birth, and birth weight (grams). We gathered each participant’s information from the forms they filled out during pre-pregnancy through the first six months of their child’s follow-up. The specific categories measured can be found in Table 4 as well as the number of participants, percent of participants with data, and the estimated hazard ratios (HRs).

Two sources of data within the ECHO program were utilized to obtain information on a depression diagnosis during pregnancy: clinical diagnosis of depression (either self-reported or medical record), and ECHO Patient-Reported Outcomes Measurement Information System (PROMIS) instruments [22]. PROMIS yields a standardized T-Score for self-reported depressive symptoms, with a mean of 50 (SD = 10). A T-score greater than 50 is indicative of more depressive symptoms than average during pregnancy, and a score below 50 indicates fewer depressive symptoms than average. These measures were restricted to pre-pregnancy and infancy life stages. Parity was calculated from harmonized ECHO data and was defined as the number of pregnancies that lasted greater than 20 weeks prior to the ECHO pregnancy of interest.

### 2.4. Statistical Methods

Descriptive statistics are presented as frequencies and relative frequencies (percent) for variables measured on a categorical scale, and as a means and standard deviations for variables measured on a continuous scale. The bivariate independent relationships between breastfeeding duration and all variables were examined. Cox’s proportional hazards models [23] were used to estimate the relative hazard of the time to breastfeeding cessation for each participant characteristic independently, as a composite multivariable adjusted model. Results from the Cox model are presented as relative HRs with the associated 95% confidence interval (CI). The variable reduction modeling approach evaluated the role, if any, of each participant characteristic as an effect measure modifier of the Food Source Quality Scale and breastfeeding duration association, followed by an evaluation of each characteristic acting as a possible confounder of the Food Source Quality Scale and breastfeeding duration association. Variable reduction for the final model was conducted by a backward elimination of the characteristics from the full model that were least significant. Further, we treated the variation due to cohorts as a random factor to account for the clustering effect by including a random effect for each cohort in the model. The plot of the survival functions was generated with baseline hazard function [24] estimated at the midpoint of the three Food Source Quality Scale categories, (i.e., 1.5, 2.5, and 3.5), and for the identified referent category for each of the covariates identified in the final model. No imputation or elimination of the missing categories was conducted. The variables that remained in our final model had no more than 25% of missingness. Imputation would not improve our results, as the relative HRs for the Food Source Quality Scale categories remained stable throughout the analysis.

## 3. Results

Of the 12,316 ECHO children with complete data available for duration of breastfeeding, 751 (6.10%) had mothers with sufficient information on maternal food questions during pregnancy to be included in analysis. 

Table 3 presents the frequencies and percentages of the covariates across the Food Source Quality categories as well as their breastfeeding durations. Mothers categorized as having High Food Source Quality tended to have a higher level of education (32.6% had a master’s or doctorate degree), a higher household income (47% reported earning >$100,000 per year) and tended to be older (43.7% were over the age of 33) compared with the other groups. More specifically, mothers in the Low Food Source Quality group had the lowest education level (52.8% reported a high school degree or some college), the lowest household income (40.7% reported earning <$30,000) and tended to be younger (70.7% were between the ages of 18–30 years old).

Table 4 presents the frequencies and percentages, means and standard deviations of breastfeeding duration, and the unadjusted relative hazard estimate and 95% CI for each category of participant characteristics considered. Thirty-six percent of the study population were observed to have High Food Source Quality, 45% had Moderate Food Source Quality, and 19% had Poor Food Source Quality. A majority of mothers identified as non-Hispanic white (43%) or Hispanic (37%). Almost half had at least a college degree and, separately, an income below $100,000. Almost half of the mothers were older than 33 years of age. Fewer than 10% gave birth at less than 37 weeks (gestational age), and 5% of births had weights less than 2500 g.

The mean duration of breastfeeding differed across the three Food Source Quality categories. Specifically, the participants with High Food Source Quality breastfed an average of 41 weeks, while the Moderate and Low-quality food groups were shorter at 27 weeks and 16 weeks, respectively.

The median duration of breastfeeding was 30 weeks for the participants with High Food Source Quality, 22 weeks for the participants with Moderate quality, and 13 weeks for those with Low Food Source Quality. As expected, maternal race/ethnicity, education, and age were associated with breastfeeding duration as well as household income, marital status, child’s gestational age at birth, and birth weight in the bivariate analysis. The mothers’ pre-pregnancy BMI and PROMIS depression score were not found to be associated with breastfeeding duration in the bivariate analysis.

Parity and maternal variables for depression and stress had large proportions of missing data. These variables were associated with breastfeeding duration but only for the category of the participants with missing values for those variables; therefore, they were eliminated in subsequent analyses.

Table 5 presents the adjusted HRs for each participant characteristic in the full model, adjusted for all characteristics (Maternal Race/Ethnicity, Maternal Education, Household Income, Marital Status, Pregnancy Depression Diagnosis, Pregnancy Depression PROMIS T-score, Parity, Maternal Age at birth, Maternal Pre-Pregnancy BMI, Gestational Age, Birth Weight) as potential confounders, and in the final model, adjusted for the characteristics found to remain relevant in the reduced model of breastfeeding duration after the variable reduction procedures. The HRs for Food Source Quality categories did not differ much between the full model and the final model where the final adjusted HRs were 0.76 for the High Food Source Quality category and 1.52 for the Low Food Source Quality category compared to the Moderate Food Source Quality category. That is, participants in the High Food Source Quality group had a 24% lower hazard of breastfeeding cessation before 6 months postpartum (95% HR CI, 0.63–0.92), and those in the Low Food Source Quality group had a 52% greater hazard of breastfeeding cessation before 6 months postpartum (95% HR CI, 1.22–1.89) compared with the Moderate group. Increased maternal education and age were significantly associated with longer breastfeeding duration. Mothers with pre-pregnancy BMI of <25 (Normal) breastfed for 37 weeks, which was 7 weeks longer than those with pre-pregnancy BMI of 25–30 (Overweight) who breastfed for 31 weeks. The HRs for the overweight group were slightly lower than for the normal group throughout the analysis. However, the difference in HRs was not statistically significant. The mothers with pre-pregnancy BMI > 30 (obese) breastfed the least, at an average of 24 weeks. Of note, fifty percent of the pre-pregnancy BMI of <25 (Normal) group were in the High Food Source Quality group.

**Figure 2:**
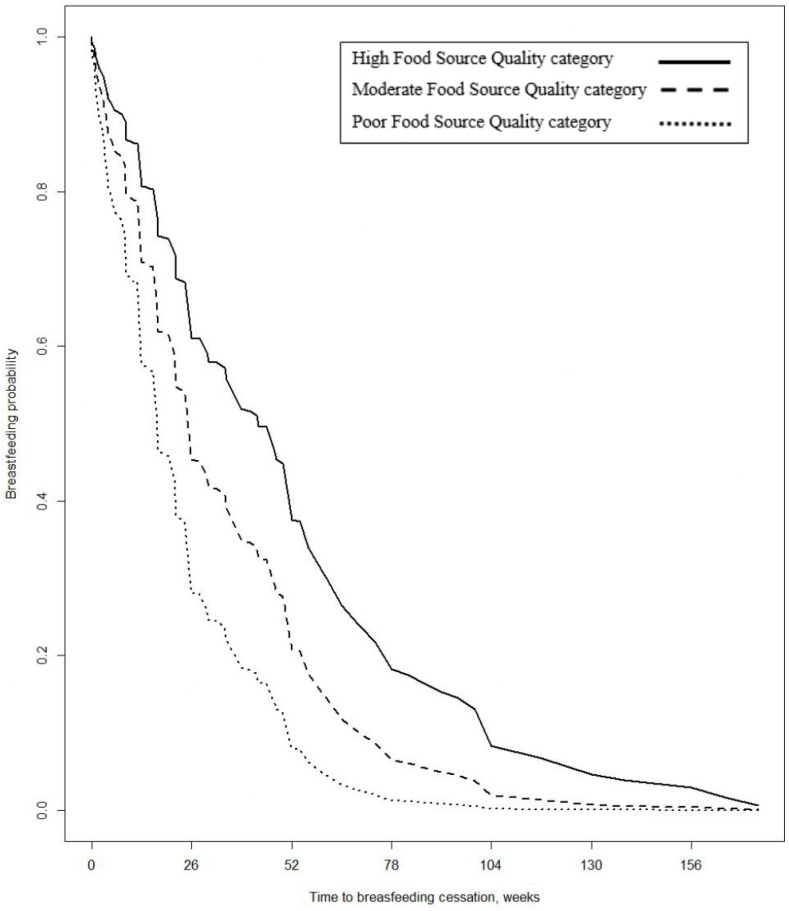
Plot of survival function (time to breastfeeding cessation) by midpoints of the three quality scale categories, adjusted for baseline hazard estimate characteristics identified in the final model.

Figure 2 depicts the survival curves for the midpoint of each of the three Food Source Quality categories adjusted for the referent group for each of the exposure variables remaining in the final model. The median time to breastfeeding cessation was 13.0 weeks for those mothers in the Low Food Source Quality category, 21.7 weeks for the Moderate Food Source Quality category, and 30.3 weeks for those with High Food Source Quality.

## 4. Discussion

The goal of this study was to examine the relationship between self-reported food source during pregnancy and breastfeeding duration, using a diverse, multi-cohort population sample. Our findings are in line with our hypothesis that mothers who reported eating more processed and fast foods and less food prepared at home breastfed for a shorter duration. More specifically, pregnant women with High Food Source Quality breastfed an average of 14 weeks longer than the Moderate Food Source Quality group and 25 weeks longer than the Low Food Source Quality group. Further, the hazard of breastfeeding cessation before 6 months postpartum for those in the High Food Source Quality group was 24% lower than the Moderate Food Source Quality group, and the Low Food Source Quality was 52% higher. This is the first time that maternal food source during pregnancy has been examined and linked to breastfeeding duration in such a large and diverse cohort.

The High Food Source Quality group consisted of women with higher education levels who were older and earning more money per year compared with the Moderate and Low Food Source Quality groups. These findings are consistent with prior research linking maternal food sources and preparation to SES and maternal age [14]. Furthermore, studies have found the SES status of consumers predicts the perceived value of organic food [25]. Indicators of low SES, such as lower education, limited income, and unemployment are associated with food insecurity in both resource-rich and resource-limited settings [26,27]. Increased maternal stress, which can be elevated in low SES communities, can be linked to poorer diets, and decreased breastfeeding duration [15]. These factors need to be prospectively examined in more detail among mother–child dyads. Taken together, these relationships are complex but highlight some of the apparent health and social inequities, the importance of education surrounding optimal nutrition during pregnancy, and the need to provide relevant resources to those in at-risk communities.

Interestingly, the data also demonstrate that mothers with the highest pre-pregnancy BMI (>30 categorized as obese) breastfed for the shortest duration. This finding is consistent with prior studies linking increased weight with poor body image, which has been linked to shorter breastfeeding duration [28]. In fact, a systematic review identified that body image concerns during pregnancy were associated with lower rates of breastfeeding intention, initiation, and shorted duration [29]. While this study only measured maternal pre-pregnancy BMI, future studies examining the effect of maternal food source, and preparation on the breastfeeding duration should also include maternal body image factors. 

The work by Gross and colleagues emphasized the importance and utility of incorporating strategies to address misconceptions about maternal diet and breast milk adequacy, managing stress, building social support networks, and connecting to supplemental nutrition assistance programs [15]. Efforts to support access to healthy fresh food and in-home cooking practices can also bolster breastfeeding duration [30]. These strategies could be implemented in obstetric clinics in at-risk communities. Maternal feeding practices greatly influence child feeding practices, and early education, resources, and interventions aimed at improving breastfeeding duration may be most useful if they start early in pregnancy when breastfeeding intention is decided [31,32]. Challenges to certain desirable eating patterns (e.g., cooking at home) during pregnancy may also be challenges to breastfeeding later.

Early maternal feeding practices are associated with children’s eating behaviors [7,8,9] and growth [9,33]. Early in infancy, data indicate that maternal weight, body image and eating concerns, concern regarding their children’s weight, and breastfeeding self-efficacy may constitute as critical barriers to exclusive breastfeeding up to 6 months [34,35]. Interestingly, prior work has demonstrated that rats fed a “junk food diet” during pregnancy and lactation predisposed offspring to obesity [36]. Taken together, maternal diet and feeding practices greatly influence early feeding patterns in the infant and child and these practices may place the child at increased risk for adverse health outcomes. It is also important to consider the context in which food is consumed. Many meal practices are deeply rooted in family traditions, which may impact factors such as food waste and mealtime practices (e.g., family meals). Future studies should examine maternal attitudes toward respect for food and eating to elucidate some of these important questions.

The scale that we used had participants’ report on their food source and preparation. We categorized the excessive usage of canned food as “poor” food source classification because fresh foods are recommended as the primary nutrient-dense dietary options and that many Americans use these products for their cost and/or convenience, and not for their nutritional properties [37]. We categorized the excessive usage of organic food as “excellent” food source classification, given that organic plants do not rely on chemical pesticide sprays to protect themselves and in turn, produce more of their own protective compounds, such as antioxidants [38]. Further, studies have also reported an increase in micronutrients such as Vitamin C, Zinc, and Iron in organic foods [38,39,40]. In the current analysis, we did not collect data on which type of canned or organic foods were consumed but rather just on the frequency. This level of detail needs to be looked at in future studies.

Our study includes several limitations. There was a high level of missingness for some covariates, making it harder to draw inference. This was a retrospective study that utilized some extant data collected before the ECHO nationwide program was officially initiated. Questionnaires collected for the ECHO nationwide program were not primarily designed to address this specific research question and as a result, the current analysis did not include important postpartum factors, such as whether mothers worked and when they returned to work, sleep, childcare support, and maternal stress. Moreover, our analysis involved the inclusion of mother–child dyads from several cohort studies that differ by measurement, visit structure, and time period. We attempted to account for this clustering effect by treating cohorts as a random effect in the modeling, but some residual confounding may remain. The maternal food source and preparation variable was designed for this study, and while it allowed us to adapt to this specific population, this may also have constituted a limitation. Using the three categories (Low, Moderate, and High) of Food Source Quality allowed clearer insights into the association between Food Source Quality and breastfeeding duration than utilizing the continuous measure alone. Our findings suggest an association between maternal food source and preparation and breastfeeding duration even when adjusting for covariates, and the support future nuanced the research examining food source and preparation.

Future work should seek to explore the relationships with other dietary factors such as maternal and infant food allergies, access to breastfeeding support, and access to parental leave from work, as these data were not available among the ECHO cohorts. Another important future direction is to explore the connections among maternal pregnancy complications, nutritional intake, and breastfeeding duration, given the emerging data linking vitamin B12 deficiency to preeclampsia [41]. Longitudinal examinations of these relationships and the way they evolve over the course of the postpartum period is also warranted.

## 5. Conclusions

Women reporting High Food Source Quality during pregnancy breastfed an average of 14 weeks longer than the Moderate Food Source Quality group and 25 weeks longer than the Low Food Source Quality group. The adjusted relative hazard ratio of time to breastfeeding cessation indicated a protective effect for the High Food Source Quality group and a detrimental effect for the Low Food Source Quality group compared with the Moderate Food Source Quality group. To our knowledge, this is the first large diverse cohort study examining the link between maternal food source and preparation during pregnancy and breastfeeding duration. We encourage future studies to examine this relationship longitudinally, with more consideration for specific details about maternal diet, food source, and preparation during pregnancy in the context of sociodemographic determinants, such as SES, that are associated with less-than-optimal food consumption.

## Figures and Tables

**Table 1:** Categorization and coding for maternal, pre-natal food source, and food preparation behaviors, Quality Score, based on the questions on the ECHO Maternal Food Source and Preparation (MFSP) questionnaire.

Food Source Questions (MFSP)	Response Options	Food Source Classification	Food Source Score
How often did you eat canned foods of any kind (meat, fish, vegetables, fruit, beans, etc.)?	Never	Excellent	1
	1 time/month	Good	2
	2–3 times/month		
	1 time/week	Average	3
	2 times/week		
	3–4 times/week		
	5–6 times/week		
	1 time/day	Poor	4
	2+ times/day		
How often did you eat fast-food or take-out food from restaurants (such as McDonalds, Chipotle, Panera, Chinese food) or prepared foods from a grocery store or deli counter?	Never	Excellent	1
	1 time/month	Good	2
	2–3 times/month		
	1 time/week	Average	3
	2 times/week		
	3–4 times/week		
	5–6 times/week		
	1 time/day	Poor	4
	2+ times/day		
How often did you eat meals that you or someone else prepared at home?	Never	Poor	4
	1 time/month	Average	3
	2–3 times/month		
	1 time/week	Good	2
	2 times/week		
	3–4 times/week		
	5–6 times/week		
	1 time/day	Excellent	1
	2+ times/day		
How often did you consume the food groups below that were organic (either fresh produce or from a jar, a package, or homemade, or labeled ‘Certified Organic’)?	Never/Rarely	Poor	4
	Sometimes	Average	3
	Often	Good	2
	Nearly always	Excellent	1

**Table 2:** Categorization of Food Source Quality mean scores.

Food Source Quality	
Mean Score *	Category
1.00–2.0	High
2.01–3.0	Moderate
3.01–4.0	Low

* Food Source Quality (Mean) Score = arithmetic mean of food source scores across the seven ECHO Maternal Food Source and Preparation (MFSP) questions per participant.

**Table 3:** Frequencies and percentages, means, and standard deviations of the covariates across the Food Source Quality categories.

Characteristics	High Food Source Quality N (%)	High Food Source Quality BF Duration Mean (SD)	Moderate Food Source Quality N (%)	Moderate Food Source Quality BF Duration Mean (SD)	Low Food Source Quality N (%)	Low Food Source Quality BF Duration Mean (SD)
**Quality scale categories**	270 (100%)	41.2 (32.9)	341 (100%)	27.2 (23)	140 (100%)	16 (16.4)
***Maternal race/ethnicity***						
Non-Hispanic White	128 (47.4%)	48.2 (34.4)	140 (41.1%)	32.6 (22.7)	52 (37.1%)	25.1 (20.7)
Non-Hispanic Black	16 (5.9%)	26.8 (26.3)	54 (15.8%)	19.8 (16.6)	20 (14.3%)	9.6 (11.9)
Hispanic	109 (40.4%)	35.3 (30)	122 (35.8%)	26 (24.9)	49 (35%)	10.9 (9.3)
Other race	<15	45.3 (34.8)	19 (5.6%)	19.2 (20)	<20	11.5 (10.5)
Unknown or missing	<5	23.8 (33.5)	6 (1.8%)	18.9 (19.3)	<5	8.8 (12.3)
***Maternal education***						
Less than high school, high school degree, GED or equivalent	31 (11.5%)	16.3 (12.1)	58 (17%)	20.5 (22.1)	37 (26.4%)	9.4 (10.2)
Some college, no degree, associate degree, trade school	49 (18.1%)	35.8 (33.4)	88 (25.8%)	25.5 (26.2)	37 (26.4%)	18.2 (16.1)
Bachelor’s degree	85 (31.5%)	49.2(37.1)	92 (27%)	31.4 (21.9)	26 (18.6%)	22.7 (19.9)
Masters, professional, or doctorate degree	88 (32.6%)	48 (28.9)	61 (17.9%)	36.3 (18.8)	8 (5.7%)	33.8 (24.6)
Unknown or missing	17 (6.3%)	27.9 (26)	42 (12.3%)	17.7 (18.3)	32 (22.9%)	11.1 (11.3)
***Household income***						
<$30,000	38 (14.1%)	25.2 (26.7)	96 (28.2%)	17.1 (20.4)	57 (40.7%)	11.8 (11.9)
$30,000–$99,999	42 (15.6%)	28.7 (19.7)	115 (33.7%)	29 (20.1)	37 (26.4%)	19.7 (18.3)
$100,000–$199,999	69 (25.6%)	50.6 (36.7)	42 (12.3%)	35.2 (23.1)	10 (7.1%)	36.3 (26.1)
$200,000 or more	58 (21.5%)	46 (27.9)	8 (2.3%)	24.6 (24.8)		
Unknown or missing	63 (23.3%)	44.6 (37.5)	80 (23.5%)	32.9 (25.6)	36 (25.7%)	13.2 (12.7)
***Marital status***						
Married or living with a partner	221 (81.9%)	43.9 (32.4)	272 (79.8%)	29.2 (23.1)	97 (69.3%)	19.2 (18.1)
Widowed, separated, divorced	5 (1.9%)	25.1 (13.5)	11 (3.2%)	30.2 (23.4)	5 (3.6%)	3.2 (1.3)
Not married, not living together	37 (13.7%)	31.4 (36.5)	45 (13.2%)	17.7 (21)	15 (10.7%)	11.1 (8.1)
Unknown or missing	7 (2.6%)	21 (18.2)	13 (3.8%)	15.7 (15.7)	23 (16.4%)	8.4 (8.9)
***Pregnancy depression diagnosis***						
No	96 (35.6%)	46.6 (34.4)	98 (28.7%)	35.8 (26.2)	40 (28.6%)	25.3 (21.2)
Yes	21 (7.8%)	53 (46.5)	27 (7.9%)	27.4 (26.1)	14 (10%)	14.6 (15.9)
Unknown or Missing	153 (56.7%)	36.2 (28.7)	216 (63.3%)	23.3 (19.8)	86 (61.4%)	11.9 (11.8)
***Pregnancy depression PROMIS T-score***						
Low score (Below 50%)	43 (15.9%)	22.4 (21.7)	117 (34.3%)	23.2 (22.1)	65 (46.4%)	10.9 (10.2)
High score (Above 50%)	34 (12.6%)	34.7 (23.7)	136 (39.9%)	23.8 (17.6)	52 (37.1%)	17.1 (16.9)
Unknown or missing	193 (71.5%)	46.6 (34.6)	88 (25.8%)	37.8 (27.9)	23 (16.4%)	27.7 (22.8)
***Parity (pregnancies > 20 weeks)***						
0	123 (45.6%)	50.2 (33.9)	39 (11.4%)	41.7 (27.1)	9 (6.4%)	28.6 (20.8)
1	39 (14.4%)	40.6 (32.4)	69 (20.2%)	29.9 (22.9)	32 (22.9%)	22.8 (19.8)
2	14 (5.2%)	45.3 (37.5)	24 (7%)	35.2 (22)	8 (5.7%)	18.5 (18.7)
3+	11 (4.1%)	41 (31.3)	15 (4.4%)	27 (33.6)	13 (9.3%)	21.1 (23.2)
Unknown or missing	83 (30.7%)	27.5 (26.2)	194 (56.9%)	22.4 (19.6)	78 (55.7%)	10.6 (9.9)
***Maternal age at birth (years)***						
18–26	50 (18.5%)	27.7 (26.1)	93 (27.3%)	19.3 (16.5)	54 (38.6%)	10.7 (8.8)
27–30	44 (16.3%)	41.7 (31.6)	90 (26.4%)	29.8 (24.5)	45 (32.1%)	16.3 (13.9)
31–33	58 (21.5%)	43.9 (31)	66 (19.4%)	33.4 (25.1)	19 (13.6%)	23.7 (26.8)
>33	118 (43.7%)	45.5 (35.5)	92 (27%)	28.3 (23.7)	22 (15.7%)	21.9 (20.2)
***Maternal pre-pregnancy BMI***						
<25	136 (50.4%)	46.3 (34.9)	80 (23.5%)	28.5 (24)	35 (25%)	20.3 (18.1)
25–30	46 (17%)	34.1 (26.4)	64 (18.8%)	36.2 (31)	36 (25.7%)	15.8 (16.6)
>30	45 (16.7%)	37.7 (35)	77 (22.6%)	20.7 (21.5)	42 (30%)	14.3 (13.2)
Unknown or missing	43 (15.9%)	36.5 (28.3)	120 (35.2%)	25.7 (15.7)	27 (19.3%)	13.3 (18.2)
***Gestational age***						
20–36 weeks	<25	28.6 (26.2)	25 (7.3%)	19.3 (17.3)	17 (12.1%)	13.5 (16.9)
37–39 weeks	139 (51.5%)	41.6 (33.1)	215 (63%)	26.3 (21.9)	81 (57.9%)	17.3 (17.2)
40+ weeks	108 (40%)	43.1 (33.6)	93 (27.3%)	30.8 (26.2)	32 (22.9%)	15.6 (15.9)
Unknown or missing	<5	46.6 (26)	8 (2.3%)	34.6 (20.4)	10 (7.1%)	10.6 (9.6)
***Birth weight (grams)***						
500–2500	11 (4.1%)	31.7 (29.6)	15 (4.4%)	20.6 (17.3)	9 (6.4%)	12.1 (17)
2501+	227 (84.1%)	45.1 (33)	282 (82.7%)	29.2 (23.5)	90 (64.3%)	18.1 (17.9)
Unknown or missing	32 (11.9%)	17.3 (19.8)	44 (12.9%)	16.5 (16.9)	41 (29.3%)	12.3 (12)

BF, breastfeeding; BMI, body mass index; GED, general education development tests; SD, standard deviation.

**Table 4:** Univariate descriptive statistics of breastfeeding duration by food source quality, participant demographic and socio-economic characteristics among 751 mother–child dyads with information available for both breastfeeding duration and mother’s food source quality scale, the bivariate estimates of association with breastfeeding duration.

Maternal and Birth Characteristics	N (%) with Data	Breastfeeding Duration, Weeks Mean (SD)	Bivariate HR Estimate	Bivariate 95% CI
**Food source quality overall**	751 (100%)	30.2 (27.6)		
***Food source quality categories***				
High	270 (36%)	41.2 (32.9)	0.8	0.66, 0.97
Moderate	341 (45%)	27.2 (23.0)	1	Referent
Low	140 (19%)	16.0 (16.4)	1.6	1.29, 1.98
***Maternal race/ethnicity***				
Non-Hispanic White	320 (43%)	37.6 (29.1)	1	Referent
Non-Hispanic Black	90 (12%)	18.8 (18.5)	1.37	1.03, 1.81
Hispanic	280 (37%)	27 (26.5)	1.14	0.93, 1.4
Other race	47 (6%)	24 (26.4)	1.39	1.01, 1.91
Unknown or missing	14 (2%)	17.4 (21.8)	1.55	0.87, 2.76
***Maternal education***				
Less than high school, high school degree, GED or equivalent	126 (17%)	16.2 (17.6)	1	Referent
Some college, no degree, associate degree, trade school	174 (23%)	26.9 (27.4)	0.67	0.53, 0.84
Bachelor’s degree	203 (27%)	37.7 (30.7)	0.55	0.43, 0.7
Masters, professional, or doctorate degree	157 (21%)	42.7 (25.8)	0.51	0.39, 0.66
Unknown or missing	91 (12%)	17.3 (18.7)	0.66	0.47, 0.92
***Household income***				
<$30,000	191 (25%)	17.1 (20.3)	1	Referent
$30,000–$99,999	194 (26%)	27.1 (19.9)	0.77	0.61, 0.98
$100,000–$199,999	121 (16%)	44.1 (32.5)	0.6	0.45, 0.81
$200,000 or more	66 (9%)	43.4 (28.3)	0.74	0.51, 1.08
Unknown or missing	179 (24%)	33.1 (30.7)	0.69	0.52,0.91
***Marital status***				
Married or living with a partner	590 (79%)	33.1 (27.8)	1	Referent
Widowed, separated, divorced	21 (3%)	22.6 (20.9)	1.01	0.65, 1.58
Not married, not living together	97 (13%)	21.9 (27.8)	1.3	1.04, 1.63
Unknown or missing	43 (6%)	12.7 (13.5)	1.57	1.08, 2.27
***Pregnancy depression diagnosis***				
No	234 (31%)	38.4 (30.1)	1	Referent
Yes	62 (8%)	33.2 (35.8)	1.17	0.88, 1.56
Unknown or missing	455 (61%)	25.5 (23.8)	1.71	1.2, 2.44
***Pregnancy depression PROMIS T-score***				
Low score (Below 50%)	222 (30%)	23.9 (19.2)	1	Referent
High score (Above 50%)	225 (30%)	19.5 (20)	1.05	0.85, 1.3
Missing	304 (40%)	42.6 (32.4)	0.79	0.57, 1.1
***Parity (pregnancies > 20 weeks)***				
0	171 (23%)	47.2 (32.3)	1	Referent
1	140 (19%)	31.3 (25.9)	1.17	0.88, 1.54
2	46 (6%)	35.4 (28.1)	1.07	0.74, 1.55
3+	39 (5%)	29 (30.1)	1.11	0.75, 1.65
Missing	355 (47%)	21 (20.6)	1.6	1.16, 2.22
***Maternal Age at birth (years)***				
18–26	197 (26%)	19 (18.9)	1	Referent
27–30	179 (24%)	29.3 (25.9)	0.71	0.58, 0.88
31–33	143 (19%)	36.4 (28.5)	0.61	0.49, 0.77
>33	232 (31%)	36.4 (31.4)	0.71	0.58, 0.88
***Maternal pre-pregnancy BMI***				
<25	251 (33%)	37 (31.5)	1	Referent
25–30	146 (19%)	30.5 (27.8)	0.92	0.75, 1.15
>30	164 (22%)	23.7 (25.9)	1.19	0.97, 1.47
Missing	190 (25%)	26.4 (20.7)	1.21	0.91, 1.62
***Gestational age***				
20–36 weeks	63 (8%)	20.9 (21.2)	1.34	1.02, 1.75
37–39 weeks	435 (58%)	29.5 (26.8)	1	Referent
40+ weeks	233 (31%)	34.4 (30.3)	0.94	0.79, 1.1
Missing	20 (3%)	23.8 (20.7)	1.14	0.69, 1.89
***Birth weight (grams)***				
500–2500	35 (5%)	21.9 (22.5)	1.41	0.99, 2
2501+	599 (80%)	33.6 (28.6)	1	Referent
Missing	117 (16%)	15.3 (16.3)	1.43	0.97, 2.1

BMI, body mass index; CI, confidence interval; GED, general education development tests; HR, hazard ratio; PROMIS, Patient-Reported Outcomes Measurement Information System; SD, standard deviation.

**Table 5:** Adjusted relative hazards of breastfeeding duration by food source quality categories from Cox’s proportional hazard model of time until breastfeeding cessation adjusting for participant demographic and socio-economic characteristics.

Maternal and Birth Characteristics	N (%)	Full Model HR Estimate	Full Model 95% CI	Final Model HR Estimate	Final Model 95% CI
**Food source quality overall**	751 (100%)				
***Food source quality categories***					
High	270 (36%)	0.75	0.62, 0.92	0.76	0.63, 0.92
Moderate	341 (45%)	1	Referent	1	Referent
Low	140 (19%)	1.46	1.16, 1.83	1.52	1.22, 1.89
***Maternal race/ethnicity***					
Non-Hispanic White	320 (43%)	1	Referent		
Non-Hispanic Black	90 (12%)	1.19	0.89, 1.6		
Hispanic	280 (37%)	1.07	0.86, 1.32		
Other race	47 (6%)	1.12	0.8, 1.58		
Unknown or missing	14 (2%)	1.41	0.75, 2.62		
***Maternal education***					
Less than high school, high school degree, GED or equivalent	126 (17%)	1	Referent	1	Referent
Some college, no degree, Associate degree, trade school	174 (23%)	0.74	0.58, 0.95	0.7	0.55, 0.9
Bachelor’s degree	203 (27%)	0.65	0.49, 0.85	0.6	0.46, 0.77
Masters, professional, or doctorate degree	157 (21%)	0.63	0.46, 0.87	0.58	0.44, 0.77
Unknown or missing	91 (12%)	0.65	0.46, 0.92	0.68	0.49, 0.95
***Household income***					
<$30,000	191 (25%)	1	Referent		
$30,000–$99,999	194 (26%)	1.03	0.79, 1.32		
$100,000–$199,999	121 (16%)	0.9	0.65, 1.26		
$200,000 or more	66 (9%)	1.18	0.77, 1.81		
Unknown or missing	179 (24%)	0.86	0.65, 1.14		
***Marital status***					
Married or living with a partner	590 (79%)	1	Referent		
Widowed, separated, divorced	21 (3%)	1.02	0.64, 1.62		
Not married, not living together	97 (13%)	1.03	0.81, 1.32		
Unknown or missing	43 (6%)	1.52	1.03, 2.26		
***Pregnancy depression diagnosis***					
No	234 (31%)	1	Referent		
Yes	62 (8%)	1.17	0.87, 1.57		
Unknown or Missing	455 (61%)	1.54	1.02, 2.31		
***Pregnancy depression PROMIS T-score***					
Low score (below 50%)	222 (30%)	1	Referent		
High score (above 50%)	225 (30%)	0.96	0.77, 1.2		
Missing	304 (40%)	0.85	0.62, 1.16		
***Parity (pregnancies > 20 weeks)***					
0	171 (23%)	1	Referent		
1	140 (19%)	1.15	0.86, 1.55		
2	46 (6%)	1.07	0.73, 1.56		
3+	39 (5%)	1.08	0.69, 1.67		
Missing	355 (47%)	1.19	0.81, 1.74		
***Maternal age at birth (years)***					
18–26	197 (26%)	1	Referent	1	Referent
27–30	179 (24%)	0.75	0.59, 0.94	0.76	0.61, 0.94
31–33	143 (19%)	0.67	0.52, 0.87	0.67	0.53, 0.85
>33	232 (31%)	0.75	0.59, 0.95	0.78	0.62, 0.97
***Maternal pre-pregnancy BMI***					
<25	251 (33%)	1	Referent	1	Referent
25–30	146 (19%)	0.9	0.72, 1.14	0.85	0.68, 1.06
>30	164 (22%)	1.2	0.96, 1.5	1.17	0.94, 1.44
Missing	190 (25%)	0.94	0.69, 1.29	1.02	0.76, 1.37
***Gestational age***					
20–36 weeks	63 (8%)	1.24	0.89, 1.74		
37–39 weeks	435 (58%)	1	Referent		
40+ weeks	233 (31%)	0.93	0.78, 1.1		
Missing	20 (3%)	1.08	0.63, 1.87		
***Birth weight (grams)***					
500–2500	35 (5%)	1.07	0.68, 1.67		
2501+	599 (80%)	1	Referent		
Missing	117 (16%)	0.99	0.66, 1.48		

BMI, body mass index; CI, confidence interval; GED, general education development tests; HR, hazard ratio; PROMIS, Patient-Reported Outcomes Measurement Information System; SD, standard deviation.

## Data Availability

The datasets for this manuscript are not publicly available because, per the NIH-approved ECHO Data Sharing Policy, ECHO-wide data have not yet been made available to the public for review/analysis. Requests to access the datasets should be directed to the ECHO Data Analysis Center, ECHO-DAC@rti.org.

## Supplementary Materials

The following supporting information can be downloaded at: https://www.mdpi.com/article/10.3390/nu14224922/s1, Table S1: Number of participants in individual cohorts.
