# Supplementary material for: Association between Quality of Maternal Prenatal Food Source and Preparation and Breastfeeding Duration in the Environmental Influences on Child Health Outcome (ECHO) Program
Source: Nutrients. 2022 Nov 21;14(22):4922. doi: 10.3390/nu14224922 (PMC9695213; doi:10.3390/nu14224922)
Supplement: Supplementary file 1 [file nutrients-14-04922-s001.zip › nutrients-1994179-supplementary.pdf]

Supplementary

Table S1. Number of participants in individual cohorts.

Cohort	Maternal Food Source and Preparation and Breastfeeding Duration
1	79 (10.5%)
2	47 (6.3%)
3	7 (0.9%)
4	128 (17%)
5	114 (15.2%)
6	34 (4.5%)
7	2 (0.3%)
8	15 (2%)
9	6 (0.8%)
10	111 (14.8%)
11	100 (13.3%)
12	108 (14.4%)
**Total**	**751**
